# The type III intermediate filament vimentin regulates organelle distribution and modulates autophagy

**DOI:** 10.1371/journal.pone.0209665

**Published:** 2019-01-30

**Authors:** Olga Biskou, Victor Casanova, Kirsty M. Hooper, Sadie Kemp, Graham P. Wright, Jack Satsangi, Peter G. Barlow, Craig Stevens

**Affiliations:** 1 School of Applied Sciences, Edinburgh Napier University, Sighthill Campus, Sighthill Court, Edinburgh, United Kingdom; 2 Centre for Genomic & Experimental Medicine, University of Edinburgh, Western General Hospital Campus, Crewe Road, Edinburgh, United Kingdom; 3 Translational Gastroenterology Unit, Nuffield Department of Medicine, John Radcliffe Hospital, Oxford, United Kingdom; Indian Institute of Science Education and Research, INDIA

## Abstract

The cytoskeletal protein vimentin plays a key role in positioning of organelles within the cytosol and has been linked to the regulation of numerous cellular processes including autophagy, however, how vimentin regulates autophagy remains relatively unexplored. Here we report that inhibition of vimentin using the steroidal lactone Withaferin A (WFA) causes vimentin to aggregate, and this is associated with the relocalisation of organelles including autophagosomes and lysosomes from the cytosol to a juxtanuclear location. Vimentin inhibition causes autophagosomes to accumulate, and we demonstrate this results from modulation of mechanistic target of rapamycin (mTORC1) activity, and disruption of autophagosome-lysosome fusion. We suggest that vimentin plays a physiological role in autophagosome and lysosome positioning, thus identifying vimentin as a key factor in the regulation of mTORC1 and autophagy.

## Introduction

Intermediate filaments (IF), along with microtubules and actin microfilaments comprise the cytoskeleton, which provides the cell with shape and structural integrity. The cytoskeleton also acts as an important framework for the modulation and control of essential cellular processes including signal transduction, the correct positioning and movement of organelles and host cell defence against infection. An important role for the cytoskeleton in the regulation of autophagy is also emerging[1, 2]. Autophagy is a tightly controlled, intracellular process that sequesters cytoplasmic material, misfolded proteins, damaged organelles and invading pathogens into double-membrane vesicles called autophagosomes, which subsequently fuse with lysosomes where content is degraded[3]. Autophagy normally occurs at a basal level, but is stimulated in response to a myriad of stresses[3]. Disruption of this pathway has been increasingly linked to a number of human diseases including neurodegeneration, cancer and inflammatory disorders[4]. Not surprisingly, there is considerable interest in exploiting autophagy for the development of novel therapies[5]. Autophagy is a complex highly dynamic process and flux through the pathways, from initial signalling events to lysosomal degradation and recycling of cellular components have been extensively reviewed[3, 6]. The pathway can however be divided into three main stages (i) early stage; pathway initiation and autophagosome formation (ii) middle stage; sequestration of content, autophagosome closure and maturation, and (iii) late stage; fusion of autophagosomes with lysosomes to form autolysosomes where content is degraded. Components of the cytoskeleton have been implicated in each stage of this process. Roles for microtubules and actin microfilaments in autophagy are already established and have been recently reviewed[7, 8]. In comparison, little is known about the role of IF proteins in the regulation of autophagy.

It is estimated there are around 70 genes in the human genome that code for IF proteins and they can be subcategorised based on similarities in amino acid sequence and protein structure[9, 10]. The type III IF, vimentin, is the most widely distributed of the IF proteins and is the major IF protein in cells of mesenchymal origin. It is abundantly expressed in various cell types including fibroblasts, endothelial cells and monocytes[11]. Vimentin structures are found throughout the cell where they are attached to organelles including the nucleus, endoplasmic reticulum, and mitochondria[11]. Early studies in yeast have demonstrated that the proper formation and distribution of autophagosomes depends on the integrity of IF networks[12], and that autophagic vacuoles were found to be tightly associated with vimentin[13, 14]. More recent studies have further linked vimentin to the regulation of autophagy in mammalian cells[15–17].

Here, using the inhibitor Withaferin A (WFA), we have investigated how vimentin regulates autophagy and report that WFA-mediated vimentin aggregation results in the relocalisation of organelles, including autophagosomes and lysosomes, from the cytosol to a juxtanuclear location. Disruption of organelle positioning inhibits mTORC1 signalling and interferes with autophagosome-lysosome fusion, resulting in an accumulation of autophagosomes that are stalled in the later stages of the autophagy pathway. Thus, vimentin is a potential molecular target for therapeutic modulation of autophagy, which is relevant to diverse human diseases.

## Materials and methods

### Cell culture, transfection and plasmids

HEK293 cells and HEK293 GFP-LC3 cells were grown in Dulbecco’s modified Eagle medium (DMEM) (Gibco, Paisley, UK) supplemented with 10% fetal bovine serum (FBS) (Invitrogen Paisley, UK) and 5x10^4^ units of penicillin streptomycin (Gibco). Cells were transfected using the Nucleofector system from Lonza using the Nucleofector Kit V (Lonza, Manchester, UK) or with Lipofectamine 2000 (Invitrogen, Waltham, MA) according to manufacturer’s instructions. The GFP-LC3[18] and GFP-RFP-LC3[19] plasmids have been described previously.

### Chemicals

Withaferin-A (WFA) (Tocris, Abingdon, UK) was diluted in dimethyl sulphoxide (DMSO) (Sigma-Aldrich, Irvine, UK) to a stock concentration of 1mg/ml. The stock solution was further diluted in DMEM to working concentrations between 0.5–10 μM. Nocodazole (NOC) (Sigma-Aldrich), was diluted in DMSO to a stock concentration of 5 mg/ml. The stock solution was further diluted in DMEM to a working concentration of 500 nM. Equivalent amounts of DMSO only were used as vehicle control. For starvation, cells were incubated in Earle’s Balanced Salt Solution (EBSS) (Gibco).

### Short interfering RNA

Cells were transfected with 200 nM On-Target*plus* Human VIM or 200 nM On-Target*plus* Human Non-targeting SMARTpool siRNA (GE Healthcare, Little Chalfont, UK) for 48 h according to the manufacturer’s instructions.

### AlamarBlue

The metabolic activity of HEK293 cells was determined using the AlamarBlue assay (Gibco). Fluorescence intensity was measured at 2, 4, 6 and 8 h with a further measurement taken at 24 h using Omega Fluostar. The excitation was set at 544 nm and the emission was measured at 590 nm. The metabolic activity of cells was calculated according to the manufacturer’s instructions.

### Antibodies

#### Primary antibodies

Rabbit anti-vimentin antibody (ab16700), rabbit anti-tubulin (ab18251), mouse anti-LAMP1 (ab25630) were from (Abcam, Cambridge, UK). Mouse anti-tubulin (T5168, Sigma-Aldrich), rabbit anti-LC3 (0260–100 LC3-2G6, Nanotools, Teningen, Germany), rpS6 (2317) and phosphorylated rpS6 (p-rpS6 S235/236, 4856) were from (New England Biolabs, Herts, UK). *Secondary antibodies for immunostaining*: anti-rabbit FITC and anti-rabbit TRIC were from (Sigma-Aldrich), anti-rabbit Alexa Fluor 647 (A21246, ThermoFisher, Paisley, UK), anti-rabbit Alexa Fluor 488 (A11034, Invitrogen), anti-mouse Alexa Fluor 568 (A11004, Invitrogen,). Actin staining was performed using Alexa Fluor 594 Phalloidin (A12381, Invitrogen,) or Alexa Fluor 488 Phalloidin (A12379, Invitrogen,). LysoTracker Red (L7528, ThermoFisher) was used to visualise lysosomes in live cell imaging. *Secondary antibodies for western immunoblotting*: goat anti-rabbit HRP (P0448, Dako, Santa Clara. USA) and goat anti-mouse HRP (P0447, Dako).

### Immunoblotting

After appropriate treatments, cells were washed and scraped in 1 x phosphate buffered saline (PBS), then lysed in ice-cold extraction buffer (50mM Tris [pH 7.6], 150mM NaCl, 5mM EDTA, 0.5% NP-40, 5mM NaF, 1mM sodium vanadate, 1 × Protease Inhibitor Cocktail (Thermofisher) for 30 min followed by centrifugation at 13,000rpm for 10 min. Protein content of cell lysates was determined using Bradford assay (Sigma-Aldrich). Samples were resolved by denaturing gel electrophoresis, typically on 10% or 15% acrylamide/bisacrylamide gels, electro-transferred to PVDF membrane (Immobilon-FL, Sigma-Aldrich) and incubated with primary antibodies overnight at 4°C. After washing, membranes were incubated with a secondary antibody for 1h at room temperature (RT). Proteins were visualised by incubation with an ECL western blotting analysis system (GE Healthcare) or Immobilon western chemiluminescent HRP substrate (Millipore, Milton, UK). Chemiluminescence was imaged using a G: BOX system (Syngene, Cambridge, UK) and where appropriate the relative intensity of bands was measured using Image J software[20]. Uncropped immunoblots are shown in (S1 Fig).

### Immunofluorescence microscopy

Cells were seeded on 21-mm borosilicate glass cover slips or 35mm imaging dishes (Ibidi, Thistle Scientific, Glasgow, UK) at a density between 7.5x 10^5^ and 9x10^5^ cells and incubated overnight to allow cells to attach.

#### For fixed cell imaging

After appropriate treatment, cells were fixed with 4% paraformaldehyde for 15 min, permeabilised with PBS/0.2% Triton X-100 (Sigma-Aldrich) and blocked with PBS containing 10% FBS or 10% goat’s serum (Gibco). For protein detection, primary antibodies were incubated in PBS/1% FBS or goat’s serum overnight at 4°C. Cells were then incubated for 1 h at RT with conjugated secondary antibodies in PBS/10% FBS or goat’s serum. Where appropriate, cells were counterstained with 4′,6′-diamidino-2-phenylindole (DAPI). Images were captured using Carl Zeiss LSM880 confocal microscope (Jena, Germany) and analysed using Image J software (National Institutes of Health, Bethesda, MD, USA).

#### For live cell imaging

After appropriate treatments, cells grown on imaging dishes were transferred to a live cell-imaging chamber attached to the microscope where optimal conditions were maintained. Images were captured using a Carl Zeiss LSM880 confocal microscope and analysed using Image J software. For quantification of confocal images, regions of interest (ROI) were drawn using the freehand selection tool to include autophagosomes, lysosomes or vimentin. The area covered in the ROI was measured using the measure tool, with values presented as bar graphs.

### Autophagy assays

#### For autophagy assays in HEK293 GFP-LC3 stable cells

The basal threshold number of GFP-LC3 puncta per cell was established as 5, and cells exhibiting greater than basal number were regarded as having enhanced autophagy activity. 20 cells were counted in 3 fields of view per experiment and the percentage cells exhibiting >5 GFP-LC3 puncta quantified.

#### For tandem fluorescent-tagged GFP-RFP-LC3 assays

Cells were transiently transfected with the GFP-RFP-LC3 plasmid and following designated treatments, the fluorescent autophagy markers RFP-LC3 or GFP-RFP-LC3 were observed using a confocal microscope and the number of puncta per cell determined.

### Flow cytometry

#### For autophagy assays in HEK293 GFP-LC3 stable cells

Cells were seeded at 3x10^5^ cells per well on a 12-well plate and incubated overnight. After treatments, cells were incubated in 0.25% trypsin at 37°C for 10 min, gently detached from the plate, re-suspended in media and collected in FACS tubes on ice. Following collection, cells were centrifuged at 300 x g for 5 min, supernatant was discarded and cells re-suspended in 0.05% w/v Saponin (Sigma-Aldrich) diluted in Dulbecco’s phosphate buffered saline (DPBS, Oxoid, Hampshire, UK) to remove the unbound cytosolic LC3. Cells were then centrifuged again as described, and the Saponin solution discarded and replaced with DPBS. Finally, cells were acquired using a BD Biosciences Celesta flow cytometer and data analysis performed using FlowJo software.

#### For Annexin V/PI staining

Cells were stained using the FITC Annexin V Apoptosis Detection Kit I (BD Pharmingen, Berkshire, UK) according to manufacturer’s instructions. Unstained cells, Annexin-V only stained cells or PI only stained cells were used to calculate voltage compensation and apply gating.

### Statistical analysis

Results are reported as the mean ±SD assuming normally distributed variables with statistical analysis conducted using unpaired t-test, one-way or two-way ANOVA as appropriate on GraphPad Prism version 7.0 for Mac (GraphPad Software, CA, USA).

## Results

### WFA causes vimentin to aggregate to a juxtanuclear location

**Fig 1:**
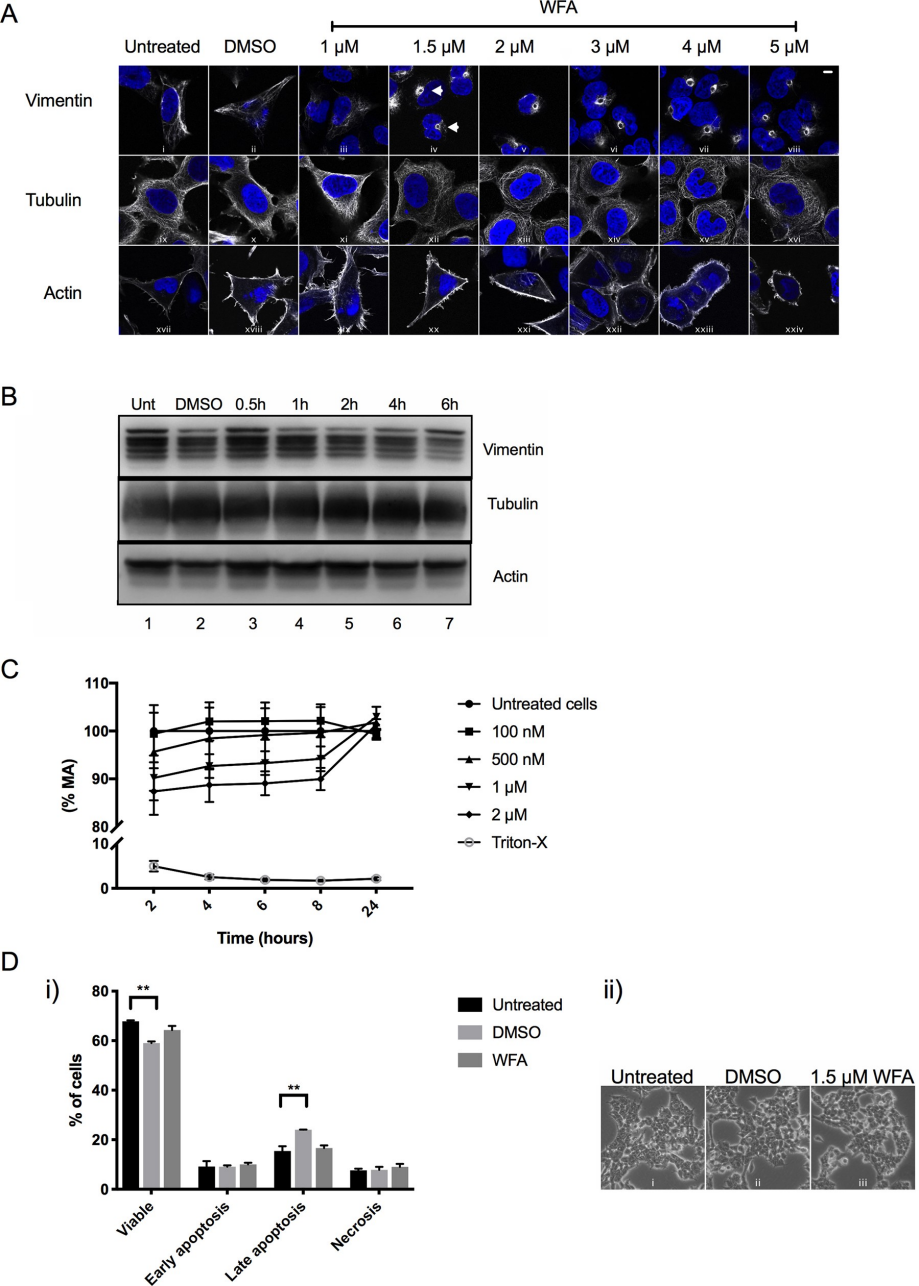
WFA causes vimentin to aggregate to a juxtanuclear location. A) Immunofluorescence analysis of endogenous vimentin, tubulin and actin (white) in HEK293 cells treated for 4 h with 1–5 μM of WFA and compared to DMSO vehicle control. Cell nuclei were stained with DAPI (blue). Representative images are shown. Scale bar is equal to 10 μm. B) Immunoblot analysis of endogenous vimentin, tubulin and actin protein levels in HEK293 cells treated for up to 6 h with 1.5 μM of WFA, DMSO vehicle control and compared to untreated cells (Unt). Because vimentin (55 kDa), tubulin (50 kDa) and actin (42 kDa) resolve at similar sizes, equal amounts of cell lysate derived from the same experiment were run simultaneously on separate gels and immunoblots were processed in parallel. Densitometry values for vimentin, tubulin and actin are presented in (S2 Fig). C) The metabolic activities of HEK293 cells were assessed using concentrations of WFA between 100 nM—2 μΜ for up to 24 h and compared to untreated cells. Triton-X was used as a positive control of toxicity. D) i) HEK293 cells were untreated or treated with DMSO vehicle control or 1.5 μΜ WFA for 6h and stained with Annexin V/PI and analysed by flow cytometry. Two-way ANOVA ** p<0.01 (n = 2 independent experiments). ii) Cell morphology was analysed by microscopy prior to flow cytometry. Brightfield images were captured at 20-x magnification. Representative images are shown.

HEK293 cells are a well-characterised cell line used in autophagy research [21]. The natural product WFA, from the plant *Withania somnifera*, binds to and inhibits vimentin [22]. To determine the effects of WFA in our experimental model, cells were treated with a range of concentrations of WFA (Fig 1A). Treatment of cells for 4 h with 1.5 μM WFA was sufficient to cause observable effects on vimentin, namely retraction of vimentin from the cell periphery and aggregation to a juxtanuclear location (Fig 1A, panel iv). Importantly, under these conditions, WFA did not disrupt tubulin or actin distribution (Fig 1A, panels xii and xx). Vimentin exists in several molecular weight forms in HEK293 cells and treatment with 1.5 μM WFA resulted in a gradual decrease in lower molecular weight forms of vimentin between 1h and 6h (Fig 1B). A ~40% decrease in total vimentin signal was observed after 6 h as quantified by densitometry (Panel A in S2 Fig). In contrast WFA had no obvious effect on protein levels or banding pattern of tubulin (Panel B in S2 Fig) or actin (Panel C in S2 Fig) at any of the time points tested. Anticancer effects of WFA have been described that include cell cycle arrest as well as cell death and apoptosis[23]. It was therefore important to test the effect of WFA on cell viability. At 1–2 μM concentrations, WFA caused a ~10% reduction in cell viability between 2 h and 8 h of treatments (Fig 1C). Furthermore, after 6 h treatment at 1.5 μM concentrations, WFA had no significant effect on early and late apoptosis or necrosis (Fig 1D, panel i) and no observable effects on cell morphology (Fig 1D, panel ii). In summary, treatment of cells with 1.5 μM WFA was sufficient to cause vimentin aggregation, while having minimal effect on other cytoskeletal components, cell morphology and cell viability.

### Vimentin inhibition causes the accumulation of LC3

**Fig 2:**
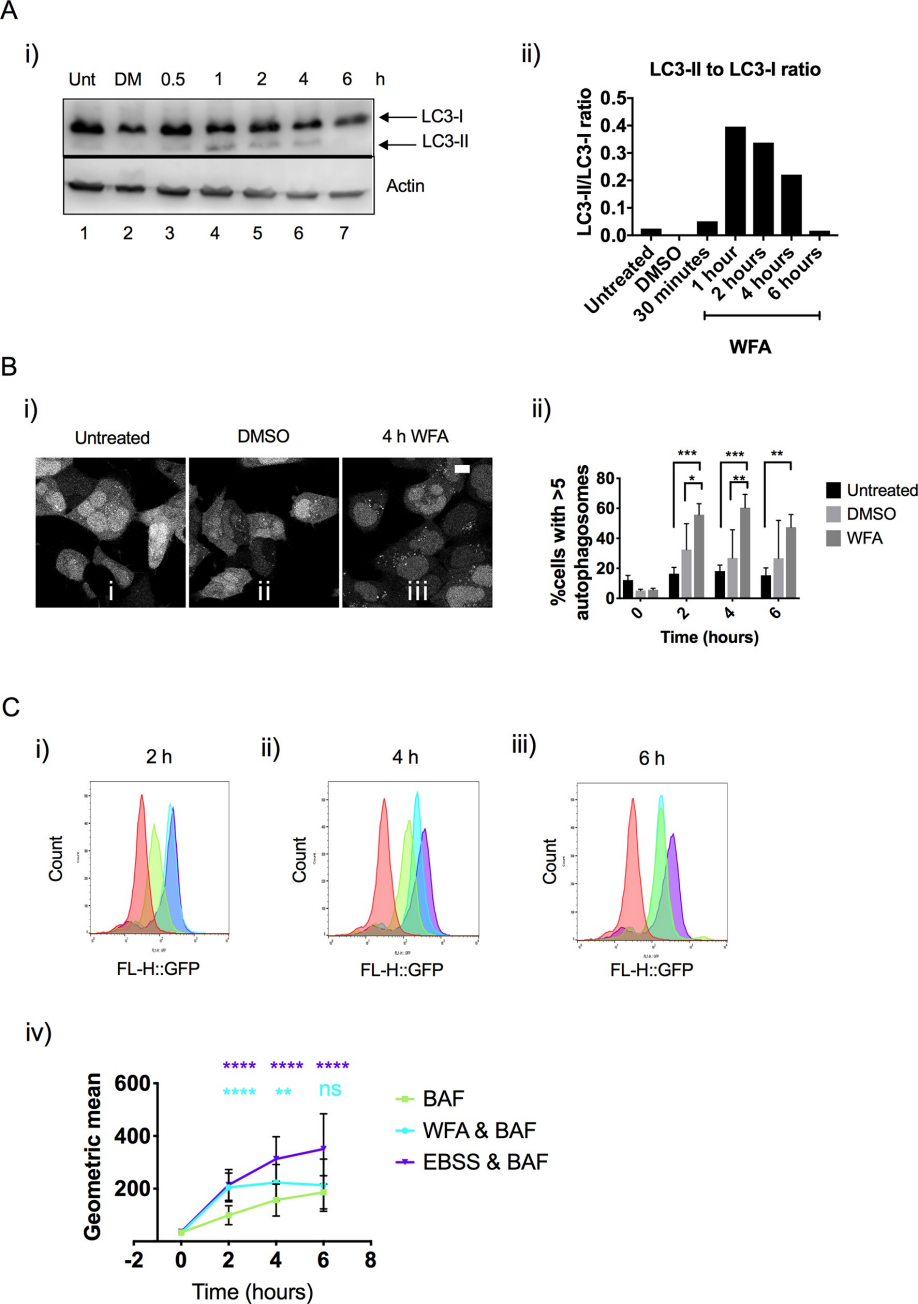
Vimentin inhibition causes the accumulation of LC3. A) i) Immunoblot analysis of endogenous LC3 in HEK293 GFP-LC3 cells treated for up to 6 h with 1.5 μM of WFA, DMSO vehicle control and compared to untreated cells (Unt). After cell lysates were resolved and transferred to membrane, the membrane was cut horizontally and incubated with antibodies specific to LC3 and actin. ii) The ratio between LC3-II and LC3-I was quantified by densitometry. B) i) Representative images from live-cell imaging of HEK293 GFP-LC3 cells treated for 4 h with 1.5 μΜ WFA, DMSO vehicle control and compared to untreated cells. ii) Quantification of cells exhibiting >5 autophagosomes at 0, 2, 4 and 6 h after treatment with 1.5 μM of WFA, DMSO vehicle control and compared to untreated cells. Two-way ANOVA ** p<0.01, ***p<0.001 (n = 3 independent experiments, 20 cells were analysed in each experiment) C) Flow cytometry analysis of HEK293 GFP-LC3 cells treated with 160nM BAF, EBSS & 160nM BAF or 1.5 μM WFA & 160nM BAF for 2 h (i), 4 h (ii) and 6 h (iii). Cells were analysed for GFP-LC3 fluorescence intensity. Representative chromatograms and quantification of the geometric means (iv) are shown. Statistical analysis compares WFA & BAF treated cells to BAF only treatment (blue stars) and EBSS & BAF treated cells to BAF only treatment (purple stars). Two-way ANOVA ns = p>0.05, ** p≤0.01, ****p≤0.0001 (n = 3 independent experiments).

To assess whether vimentin regulates autophagy, we utilised HEK293 cells stably expressing the autophagy marker LC3 fused to green fluorescent protein (HEK293 GFP-LC3). Immunoblot analysis of cells treated with WFA showed the accumulation of endogenous LC3-II after 1 h of treatment and this could be observed up to 4 h post treatment (Fig 2A, panel i, lanes 4–6) and quantified in (Fig 2A, panel ii). As a further means of testing the effects of vimentin inhibition on autophagy we used live cell imaging. In cells treated with WFA, the accumulation of GFP-LC3 containing autophagic puncta was clearly observed and peaked after 4 h (Fig 2B, panel i) and quantified in (Fig 2B, panel ii). Cells were also analysed for accumulation of GFP-LC3 using flow cytometry. To optimise flow cytometric analysis, cells were washed with the glycoside Saponin to permeabilise cell plasma membranes prior to analysis. Plasma membrane permeabilisation is known to release unbound cytosolic LC3 with only the lipidated form of LC3-II, which is tightly associated with autophagosome membranes, being retained. In addition, experiments were conducted in the presence of a low concentration of BAF to allow assessment of autophagic flux. After 2 h, BAF treatment resulted in an increased accumulation of GFP-LC3-II compared to the untreated control (Fig 2C, panel i), and the WFA & BAF and EBSS & BAF treatments resulted in a greater accumulation of GFP-LC3-II compared to the BAF only treatment, suggesting that inhibition of vimentin stimulates autophagic activity. After 4 h, WFA & BAF and EBSS & BAF treatments still resulted in a greater accumulation of GFP-LC3-II compared to BAF only (Fig 2C, panel ii), again suggesting that inhibition of vimentin was stimulating autophagic activity. In contrast, after 6 h, WFA & BAF resulted in an accumulation of GFP-LC3-II which was similar to BAF only, and was lower when compared to EBSS & BAF (Fig 2C, panel iii). Quantification of the geometric means over time is shown (Fig 2C, panel iv). In summary, at early time points inhibition of vimentin causes LC3-II to accumulate and appears to be stimulating autophagy, however at later time points inhibition of vimentin becomes indistinguishable from BAF only. This is in contrast to EBSS, a potent stimulator of autophagy, which clearly drives the accumulation of LC3-II positive autophagosomes to levels in excess of BAF only. It was also important to compare pharmacological inhibition of vimentin with short interfering RNA (siRNA)-mediated silencing of gene expression. Gene silencing effectively depleted vimentin protein levels (Panel A in S3 Fig), however there were no observable effects on basal autophagy levels (Panel B in S3 Fig) suggesting that depletion of vimentin from the cell and aggregation of vimentin have disparate effects on autophagy.

### Vimentin inhibition causes the juxtanuclear clustering of autophagosomes

**Fig 3:**
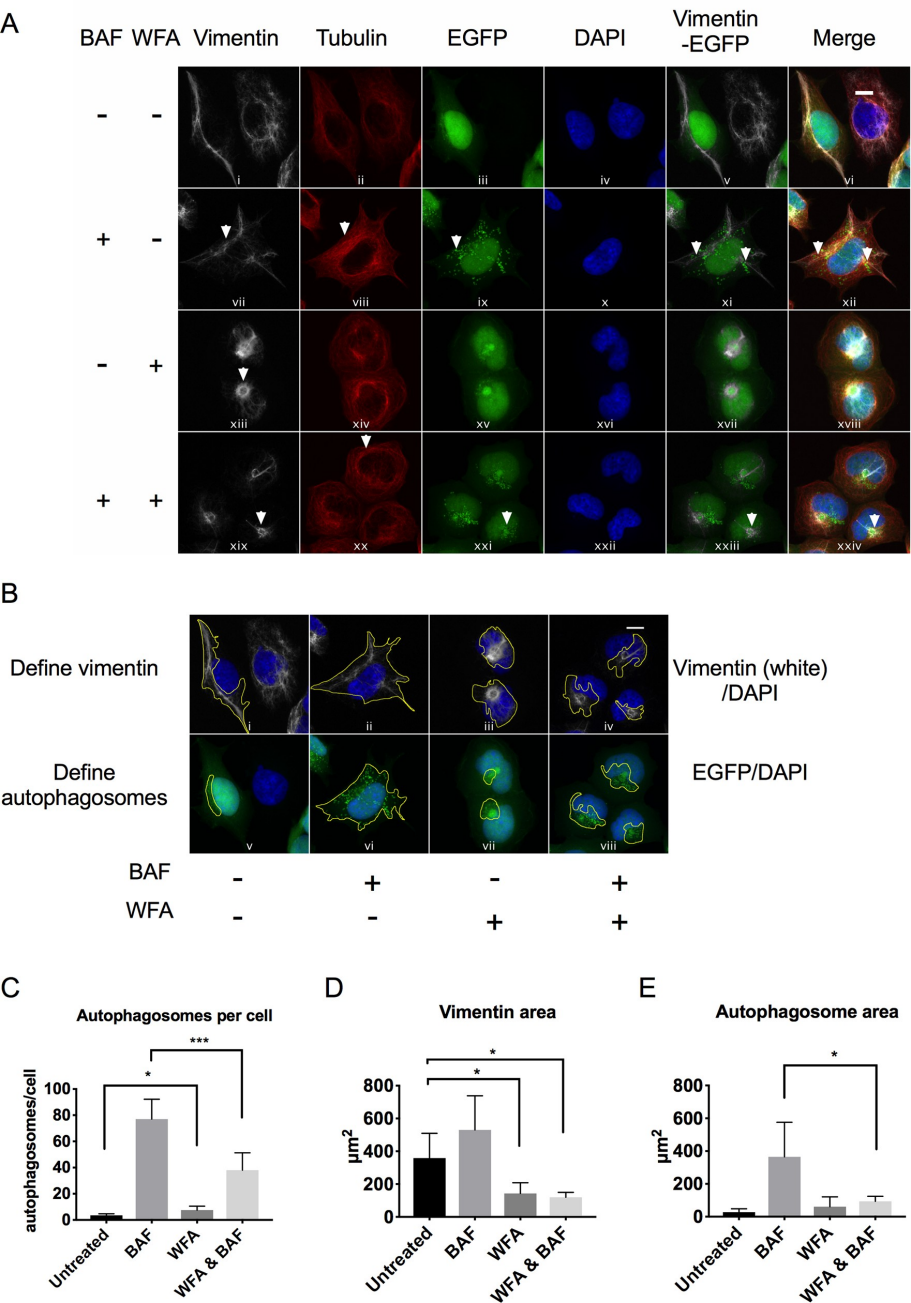
Vimentin inhibition causes the juxtanuclear clustering of autophagosomes. A) Immunofluorescence analysis of endogenous vimentin (white), tubulin (red) and GFP-LC3 in HEK293-GFP-LC3 cells treated with 160nM BAF only, 1.5 μM WFA or 1.5 μM WFA & 160nM BAF for 6 h and compared to untreated cells. Cell nuclei were stained with DAPI (blue). Merged panels show the position of GFP-LC3 relative to vimentin, tubulin and nuclei. Scale bars are equal to 10 μm. Representative images are shown. B) Yellow lines define the areas covered by vimentin (panels i-iv) or autophagosomes (panels v-viii). Scale bars are equal 10 μm. Representative images are shown. C) Number of autophagosomes per cell quantified from immunofluorescent images (n = 6 cells). Student’s t-test * p = 0.0144, *** p = 0.0008. D) Vimentin area quantified from immunofluorescent images (n = 6 cells). One-way ANOVA * p ≤0.05. E) Autophagosome area quantified from immunofluorescent images (n = 6 cells). Student’s t-test * p = 0.011.

It has been reported previously that autophagic vacuoles are found to be tightly associated with vimentin[13, 14]. To explore how vimentin inhibition affects autophagy we treated cells with BAF to first accumulate autophagosomes, and then assessed the effect of WFA on autophagosome distribution by confocal microscopy. BAF only treatment resulted in accumulation of autophagic puncta (Fig 3A, panel ix). Consistent with our previous observations, WFA treatment caused vimentin to aggregate to a juxtanuclear location (Fig 3A, panels xiii and xix) with minimal effects on tubulin distribution (Fig 3A, panel xiv and xx). Autophagosomes redistributed from the cytosol to a juxtanuclear location upon WFA treatment (Fig 3A, panel xv) or BAF and WFA treatment (Fig 3A, panel xxi), in close proximity with vimentin (Fig 3A, panel xxiii). Quantification of our results show that WFA treatment caused a significant reduction in autophagosome number, due to difficulties counting individual autophagosomes (Fig 3C). To quantitatively estimate the extent of vimentin and autophagosome clustering at the juxtanuclear region of a cell, we adopted a method described by Erie *et al*[24] (Fig 3B). Quantification shows that WFA caused a significant reduction in the area occupied by vimentin (Fig 3D) and autophagosomes (Fig 3E). In addition, we assessed the effect of siRNA-mediated silencing of vimentin gene expression on autophagosome distribution. Gene silencing effectively depleted vimentin protein levels and BAF treatment accumulated autophagosomes, however there were no observable effects on autophagosome distribution in vimentin depleted cells (Panel A in S4 Fig). These results confirm that depletion of vimentin from the cell and aggregation of vimentin have disparate effects on autophagy.

### Effect of microtubule inhibition on vimentin and autophagosome distribution

**Fig 4:**
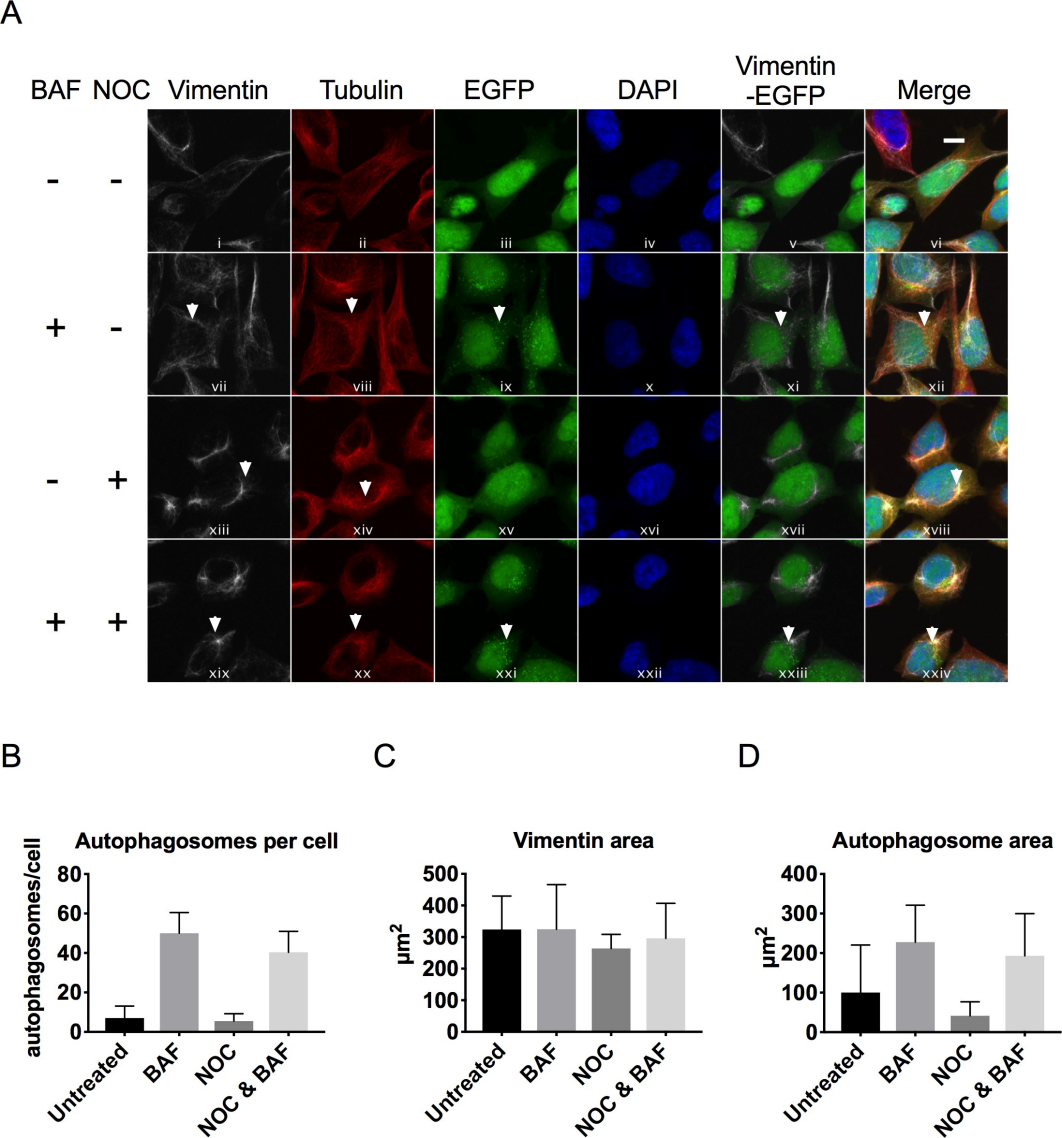
Effect of microtubule inhibition on the distribution of vimentin and autophagosomes. A) Immunofluorescence analysis of endogenous vimentin (white), tubulin (red) and GFP-LC3 in HEK293-GFP-LC3 cells treated with 160nM BAF, 500 nM NOC or 500 nM NOC & 160nM BAF for 6 h and compared to untreated cells. Cell nuclei were stained with DAPI (blue). Merged panels show the position of GFP-LC3 relative to vimentin, tubulin and nuclei. Scale bars are equal to 10 μm. Representative images are shown. B) Number of autophagosomes per cell quantified from immunofluorescent images (n = 6 cells). C) Vimentin area quantified from immunofluorescent images (n = 6 cells). D) Autophagosome area quantified from immunofluorescent images (n = 6 cells).

It has also been reported that microtubules interact with autophagosomes and play a role in their cellular localisation[7]. To explore the effect of microtubule inhibition on vimentin and autophagosome distribution we treated cells with BAF to first accumulate autophagosomes, and then assessed the effect of the microtubule polymerisation inhibitor nocodazole (NOC) by confocal microscopy. BAF only treatment resulted in accumulation of autophagic puncta (Fig 4A, panel ix). NOC treatment caused tubulin to adopt a more compacted distribution (Fig 4A, panel xiv and xx) and some effect on vimentin distribution was also observed (Fig 4A, panel xiii and xix). In contrast to WFA treatment, autophagosomes did not accumulate in response to NOC (quantified in Fig 4B), and remained distributed throughout the cytosol (Fig 4A, panel ix and xxi). Furthermore, NOC treatment did not have a significant effect on the area occupied by vimentin (Fig 4C) or autophagosomes (Fig 4D). Taken together, these results suggest that vimentin plays a major role in regulating autophagosome positioning within the cell.

### Vimentin inhibition causes the juxtanuclear clustering of lysosomes

**Fig 5:**
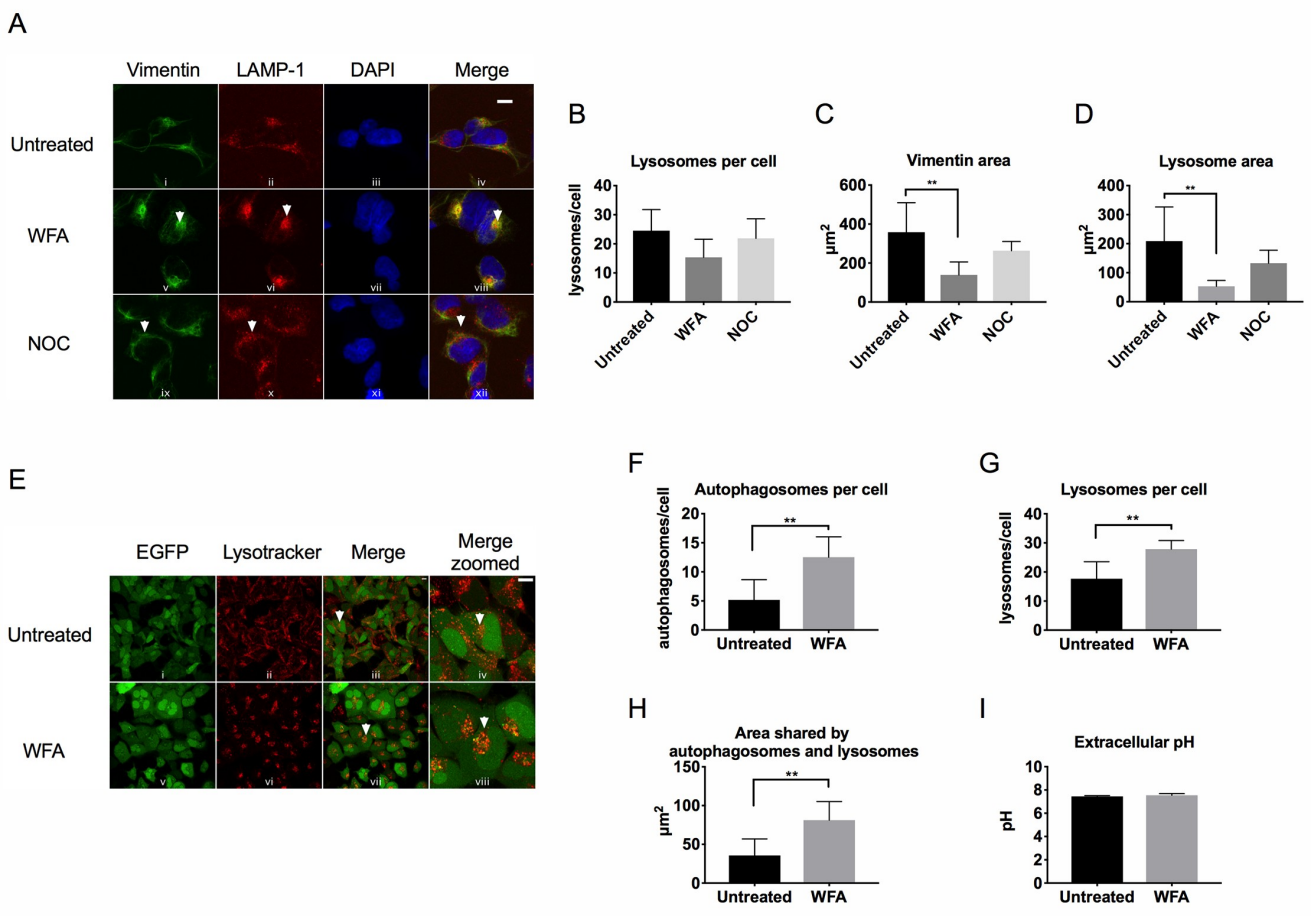
Vimentin inhibition causes the juxtanuclear clustering of lysosomes. A) Immunofluorescence analysis of endogenous vimentin (green) and endogenous LAMP-1 (red) in HEK293 cells treated for 6 h with 1.5 μM of WFA or 500 nM NOC and compared to untreated cells. Cell nuclei were stained with DAPI (blue). In the merged panels, areas where vimentin and LAMP1 colocalise appear as yellow. Scale bars are equal to 10 μm. Representative images are shown. B) Number of lysosomes per cell quantified from immunofluorescent images (n = 6 cells). C) Vimentin area quantified from immunofluorescent images (n = 6 cells). One-way ANOVA **p = 0.0032. D) Lysosome area quantified from immunofluorescent images (n = 6 cells). One-way ANOVA ** p = 0.0044. E) Live cell imaging analysis of GFP-LC3 and lysosomes (stained with Lysotracker Red) in HEK293-GFP-LC3 cells treated with 1.5 μM WFA for up to 6 h and compared to untreated cells. In the merged panels, areas where GFP-LC3 and lysosomes colocalise appear as yellow. Representative images of 6 h treatment is shown. Scale bars are equal to 10 μm. F) Number of autophagosomes per cell quantified from live cell imaging (n = 6 cells). Student’s t-test **p = 0.0046. G) Number of lysosomes per cell quantified from live cell imaging (n = 6 cells). Student’s t-test **p = 0.0036. H) Area shared by autophagosomes and lysosomes quantified from live cell imaging (n = 6 cells). Student’s t-test **p = 0.0059. I) Extracellular pH measurements from HEK293-GFP-LC3 cells treated with 1.5 μM WFA for 6 h and compared to untreated control.

Vimentin is attached to numerous organelles within the cell including the nucleus, endoplasmic reticulum and mitochondria[11]. In addition, lysosome distribution is altered in cells lacking IF [25]. To explore how vimentin inhibition affects lysosomes we used HEK293 cells and monitored lysosome distribution via immunofluorescence staining of endogenous lysosomal membrane-associated protein 1 (LAMP1). WFA treatment caused vimentin (Fig 5A, panel v) and lysosomes (Fig 5A, panel vi) to aggregate to a juxtanuclear location with colocalisation clearly observed (Fig 5A, panel viii). In contrast, NOC treatment had no observable effect on the distribution of vimentin (Fig 5A, panel ix) or lysosomes (Fig 5A, panel x). Quantification of our results show that WFA treatment caused a non-significant reduction in lysosome number, due to difficulties counting individual lysosomes (Fig 5B). However, a significant reduction in the area occupied by vimentin (Fig 5C) and lysosomes (Fig 5D) was observed. As an additional test, we used live cell imaging. Cells were incubated with Lysotracker Red, treated with WFA and both GFP-LC3 puncta and lysosome distribution were recorded in real time. Consistent with our previous observations, WFA treatment caused autophagosomes (Fig 5E, panel v) and lysosomes (Fig 5E, panel vi) to accumulate in a juxtanuclear location. Importantly, upon WFA treatment we observed only limited colocalisation of autophagosomes with lysosomes (Fig 5E, panel viii). Quantification shows that the number of autophagosomes (Fig 5F), the number of lysosomes (Fig 5G) and the area shared by autophagosomes and lysosomes (Fig 5H) are all significantly increased upon WFA treatment.

The pH of extracellular cell culture media has been shown to affect the intracellular trafficking of lysosomes[26]. To determine whether WFA affects pH, media was sampled under the same conditions as the live cell imaging analysis, and our results revealed no change upon treatment with WFA (Fig 5I). As an additional control we also assessed the effect of vimentin inhibition on the distribution of mitochondria. Cells were incubated with Mitotracker Red, treated with WFA, and mitochondrial distribution determined by confocal fluorescent microscopy. Consistent with our previous observations, WFA treatment caused vimentin and mitochondria to accumulate to a juxtanuclear location (S5 Fig). Taken altogether, our results indicate that vimentin plays a key role in regulating the distribution of organelles within the cell.

### Vimentin network aggregation modulates mTORC1 activity

**Fig 6:**
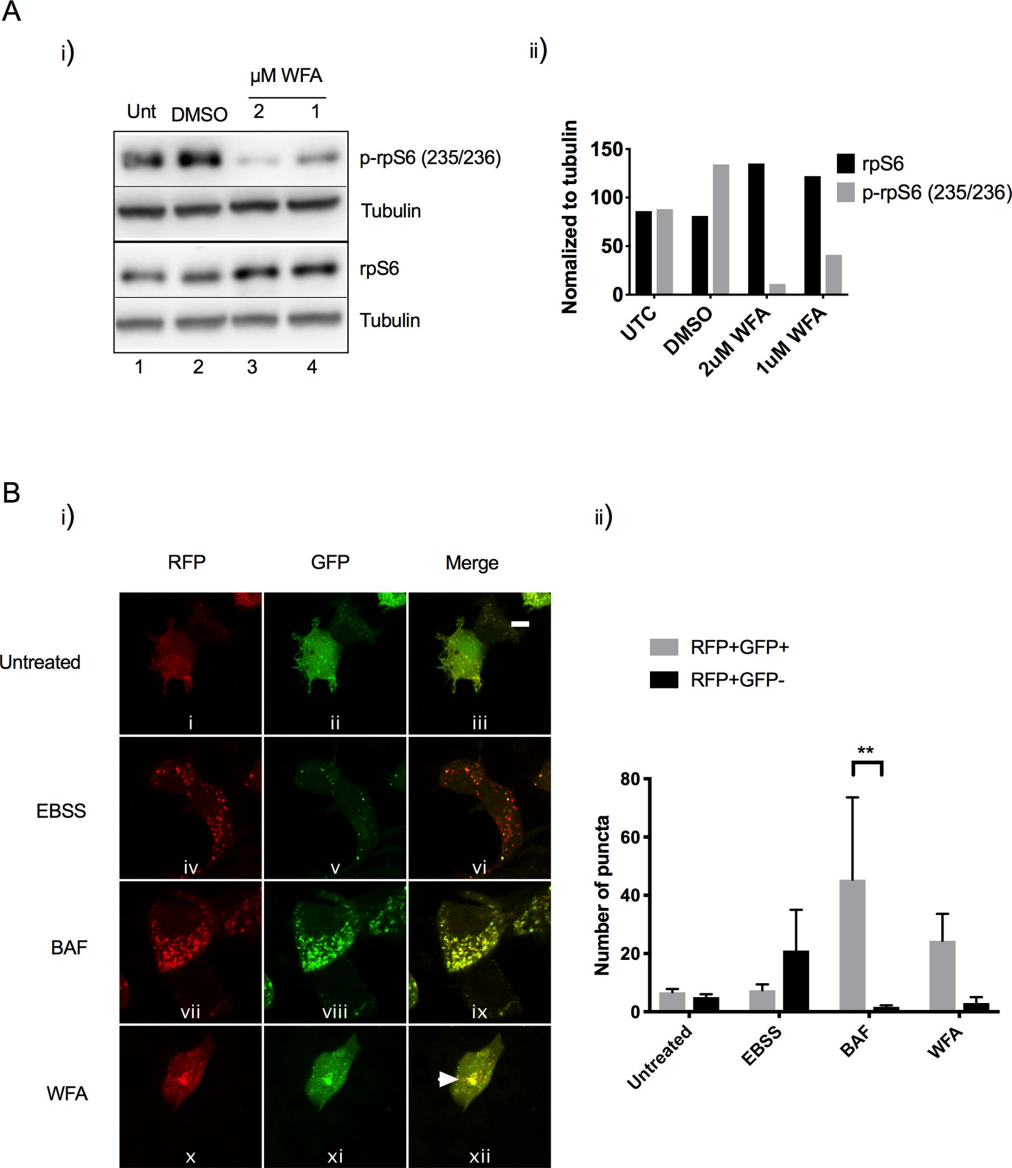
Vimentin inhibition modulates mTORC1 activity and fusion of autophagosomes and lysosomes. A) i) HEK293 cells were untreated or treated with DMSO, 1μM WFA or 2μM WFA for 6 h. Protein lysates were immunoblotted for endogenous rpS6, phosphorylated rpS6 (p-rpS6 (S235/236)) and tubulin. ii) The ratio of rpS6/p-rpS6 (normalized to tubulin) was quantified by densitometry. B) i) Immunofluorescence analysis of HEK293 cells transiently transfected with the GFP-RFP-LC3 plasmid and treated with EBSS, 160nM BAF or 1.5 μM of WFA for 6 h. Change of both green and red fluorescence was assessed by confocal microscopy. The numbers of acidified autophagosomes (RFP+GFP-) versus neutral autophagosomes (RFP+GFP+) per cell in each condition were counted. (n = 3 cells). Scale bars are equal to 10 μm. ii) Quantification of acidified autophagosomes (RFP+GFP-) versus neutral autophagosomes (RFP+GFP+).

Mechanistic target of rapamycin complex 1 (mTORC1) is a master regulator of cell growth and a potent inhibitor of autophagy[27] therefore we reasoned that inhibition of vimentin by WFA may affect mTORC1 activity. mTORC1 controls protein translation via phosphorylation of p70-S6 kinase 1 (S6K1) and ribosomal protein S6 (rpS6), which can be used as surrogate markers of mTORC1 activity. Immunoblotting revealed that WFA treatment caused a dramatic decrease in p-rpS6 in a dose dependent manner (Fig 6A, panel i, lanes 3 and 4). The ratio of p-rpS6: total rpS6 is quantified in (Fig 6A, panel ii). These results indicate that an intact vimentin network is important for mTORC1 activity.

### Vimentin network aggregation modulates fusion of autophagosomes and lysosomes

To investigate whether inhibition of vimentin modulates the fusion of autophagosomes and lysosomes, we employed a tandem GFP-RFP-LC3 plasmid[19]. This GFP-RFP-LC3 plasmid utilises the pH difference between the acidic autolysosome (formed by fusion of an autophagosome and lysosome) and the neutral autophagosome, with the pH sensitivity differences exhibited by GFP (labile at acidic pH) and RFP (stable at acidic pH). Thus, this plasmid can be used to monitor maturation of the autophagosome (RFP+GFP+) to the autolysosome (RFP+GFP-). To allow confocal image-based analysis, cells were transiently transfected with the plasmid prior to treatment with WFA and compared to BAF or EBSS that acted as controls. Upon EBSS treatment, an accumulation of (RFP+GFP-) autophagic puncta was observed; indicating that complete progression through the pathway was taking place (Fig 6B, panel iv, v and vi). BAF treatment resulted in the accumulation of (RFP+GFP+) puncta with almost complete colocalisation as indicated by yellow puncta, indicating that fusion between autophagosomes and lysosomes was not taking place (Fig 6B, panel vii, viii and ix). WFA treatment also resulted in the accumulation of (RFP+GFP+) puncta with almost complete colocalisation as indicated by yellow puncta (Fig 6B, panel x, xi and xii) suggesting that inhibition of vimentin has an effect similar to BAF, blocking the maturation of autophagosomes to autolysosomes. Significantly, (RFP+GFP+) puncta clustered to a juxtanuclear location in response to WFA treatment (Fig 6B, panel x, xi and xii). These results suggest that inhibition of vimentin can modulate progression through the autophagy pathway by relocalising autophagosomes from the cytosol to a juxtanuclear location.

## Discussion

Here we report a positive role for vimentin in the regulation of autophagy. Our initial results by immoblotting and live-cell imaging indicated that pharmacological inhibition of vimentin by WFA caused an accumulation of LC3-II and autophagosomes, suggesting that vimentin acts to inhibit autophagy. However, further investigation by flow cytometry demonstrated that WFA disrupts progression through the autophagy pathway and acts similar to the autophagy inhibitor BAF. Our results are in agreement with recent studies showing that WFA treatment increased autophagosome accumulation but blocked the degradation of autophagic cargo[15, 16]. Importantly, our study is the first to link the effect of WFA on autophagy directly to vimentin. We also evaluated inhibition of vimentin by siRNA-mediated silencing of gene expression. In contrast to WFA, siRNA-mediated depletion of vimentin had no observable effect on basal autophagy levels or on autophagosome distribution. This is likely due to the fundamental difference in their mechanisms of action; siRNA depletes vimentin from the cell, whereas WFA causes vimentin to aggregate. Our results suggest that while WFA-inhibited vimentin may lack activity, it can still interact with binding partners including autophagosomes and lysosomes.

Immunofluorescent staining revealed that while autophagosomes and lysosomes were in close proximity after WFA treatment, they did not appear to colocalize. Furthermore, results with the GFP-RFP-LC3 plasmid demonstrate that the fusion between autophagosomes and lysosomes does not occur properly in the presence of WFA. Although we lack evidence to support the hypothesis at this time, we suggest that vimentin may also interact with and facilitate proteins that mediate fusion between autophagosomes and lysosomes. Proteins that are involved in fusion between autophagosomes and lysosomes have been recently reviewed[28], with only three groups of proteins confirmed to interact with vimentin. The microtubule plus end motor proteins, kinesins, and the minus end motor proteins dynein and dynactin have been shown to directly interact with both soluble and filamentous vimentin[29]. The effect of WFA on the motor proteins has not been investigated, however it has been observed that loss of kinesin function results in a juxtanuclear clustering of vimentin[30]. Vimentin has also been shown to interact with the small GTPase Rab7a, one of the key proteins that facilitates autophagosome and lysosome fusion[31, 32]. As with motor proteins, the effect of WFA on Rab7a is currently unknown, however it has been suggested that Rab7a promotes vimentin re-arrangements via a phosphorylation dependent mechanism[31]. Additionally, it has been suggested that WFA can inhibit SNARE-mediated fusion of autophagosomes and lysosomes[16], however the role of vimentin in this context is not understood. Furthermore, knowledge around vimentin mediated regulation of late endosomal and lysosomal positioning is sparse, and clearly further studies are required to investigate the function of known regulators of endosome and lysosome positioning upon vimentin inhibition.

A role for microtubules and actin in trafficking of autophagosomes and lysosomes has already been proposed[7, 8]. Of relevance to this study, microtubules can traffic autophagosomes from peripheral locations in the cell towards the microtubule organising centre (MTOC), a major site of microtubule nucleation located near the nucleus. The MTOC is known to be a site where lysosomes can cluster, thus facilitating autophagosome-lysosome fusion[7]. Microtubules and actin are in constant communication and linked together by cytolinkers[33] and evidence suggests there are also intimate interactions between vimentin and microtubules[34, 35]. In attempt to distinguish between the role of microtubules and vimentin in autophagosome and lysosome trafficking, we used the lowest concentration of WFA capable of aggregating vimentin, with the minimum possible effect on microtubules and actin. However, we note that WFA may affect microtubules and actin in our studies, nonetheless, we suggest that our data clearly demonstrates a key role for vimentin in autophagosome and lysosome trafficking.

Importantly, lysosomes are also the main sites for activation of mTORC1. mTORC1 is recruited to the lysosomal membrane where it is activated by Ras homology enriched in brain (Rheb), however, when autophagy is activated, for example during nutrient deprivation, mTORC1 is released from lysosomes and becomes inactivated[36, 37]. Significantly, lysosome positioning has been shown to play an important role in modulating mTORC1 activity[38] and lysosome distribution is altered in cells lacking IF [25]. Our results are in agreement with these previous studies and reveal a novel role for vimentin in the modulation of mTORC1 that has far reaching implications beyond the regulation of autophagy.

Previous work from our group demonstrated that vimentin interacts with the pattern recognition receptor (PRR) nucleotide-binding oligomerisation domain-containing protein 2 (NOD2), a bacterial sensor protein that is strongly associated with inflammatory bowel disease Crohn’s disease (CD)[39, 40]. We showed that vimentin regulates NOD2 activities including nuclear factor-kappaB (NF-κB) activation and handling of CD-associated adherent invasive strains of *Escherichia coli* (AIEC). Significantly, we also identified association of a single-nucleotide polymorphism in VIM with CD susceptibility[40]. Modification of the host cell cytoskeleton by bacterial pathogens to facilitate infection is well characterised and interest in the role of vimentin in response to bacterial infection is now growing[41]. Vimentin is expressed on the surface of certain cell types where it can act as an attachment receptor for bacteria facilitating pathogen entry into cells. The list of viruses associated with vimentin is also rapidly growing[41]. Vimentin may also play a direct role in regulating pathogen dissemination within the cytosol of host cells. Cytoplasmic bacteria, such as *Shigella flexneri* can be trapped in septin cages and compartmentalised, restricting motility and promoting clearance by autophagy[42, 43]. Vimentin cages can form around *Salmonella*-containing vacuoles[44] and *Chlamydia trachomatis* resides in membrane-bound vacuoles surrounded by a cytoskeletal network including vimentin[45], however whether this is part of the host defence response, or linked to autophagy, has yet to be determined. Interestingly, vimentin also forms a cage around the aggresome, an aggregation of misfolded proteins formed upon ubiquitin proteasome system (UPS) failure[46], and proteasome inhibitors promote clustering of lysosomes around the aggresome to facilitate degradation of the misfolded proteins by the autophagy-lysosome pathway[47]. Clearly, the system is complex, and requires further work to determine how vimentin cages, the UPS and autophagy functionally intersect.

Vimentin is also secreted from activated macrophages in response to pro-inflammatory signals[48]. Pro-inflammatory cytokine tumour necrosis factor-alpha (TNF-α) increases vimentin production and secretion, and conversely, the anti-inflammatory cytokine Interleukin-10 (IL-10) inhibits this process. The function of secreted vimentin is not known, however it is tempting to speculate that it may function similar to neutrophil extracellular traps (NETs) to trap and kill pathogens[49]. Importantly, some pathogens have evolved to inactivate vimentin. For example, vimentin is down regulated in macrophages infected with *Mycobacterium tuberculosis*, contributing to increased replication and persistence of the pathogen within these cells, confirming the importance of vimentin in host cell response to infection[50]. Cytosolic vimentin also plays a role in mediating additional innate immune responses. Activation of the NLRP3 inflammasome and subsequent maturation of the pro-inflammatory cytokine IL-1β is dependent on direct protein-protein interaction between NLRP3 and vimentin[51], suggesting that vimentin acts as a platform for assembly of the inflammasome protein complex.

To conclude, results from our own studies together with recent evidence in the literature suggest that vimentin acts as a scaffold to coordinate host immune defence processes including PRR localisation, inflammasome activation and autophagic signalling. In addition to its role in CD and host response to infection, vimentin has been linked with a growing number of diseases, including cancers, cataracts, rheumatoid arthritis and atherosclerosis [52]. Therefore, better understanding of the biology and functions of vimentin may identify novel druggable targets and shape the development of a new generation of therapeutics.

## Supporting information

S1 Fig Uncropped immunoblots. Uncropped immunoblots of vimentin (A), tubulin (B) and actin (C) corresponding to (Fig 1B); LC3-I/LC3-II (D) and actin (E) corresponding to (Fig 2A); and p-rpS6 (235/236), total rpS6 and tubulin (F) corresponding to (Fig 6A). Red boxes indicate where images were cropped.
(TIFF)

S2 Fig Quantification of vimentin, tubulin and actin protein levels. The relative intensity of vimentin (A), tubulin (B) and actin (C) was measured using Image J software.
(TIF)

S3 Fig Effect of siRNA VIM on basal autophagy. A) Immunofluorescence analysis of endogenous vimentin (red) and autophagosomes (green) in HEK293 GFP-LC3 cells treated for 48 h with 200 nM of human VIM or 200 nM human Non-targeting siRNA and compared to untreated cells. Scale bars are equal to 10 μm. Representative images are shown.
B) Quantification of cells exhibiting >5 autophagosomes after 48 h of siRNA treatment and compared to untreated cells.
(TIFF)

S4 Fig Effect of siRNA VIM on autophagosome distribution. A) Immunofluorescence analysis of endogenous vimentin (red) and autophagosomes (green) in HEK293 GFP-LC3 cells treated for 48 h with 200 nM of human VIM or 200 nM human Non-targeting siRNA followed by BAF for 6 h and compared to BAF only treated cells. Scale bars are equal to 10 μm. Representative images are shown.
B) Quantification of cells exhibiting >5 autophagosomes after 48 h of siRNA and BAF treatment.
(TIFF)

S5 Fig Effect of vimentin inhibition on mitochondria distribution. Immunofluorescence analysis of endogenous vimentin (green) and mitochondria (Mitotracker red) in HEK293 cells treated for 6 h with 1.5 μM of WFA, DMSO and compared to untreated cells. Cell nuclei were stained with DAPI (blue). Scale bars are equal to 10 μm. Representative images are shown.
(TIFF)
