# Supplementary figures and images for: The type III intermediate filament vimentin regulates organelle distribution and modulates autophagy
Source: PLoS One. 2019 Jan 30;14(1):e0209665. doi: 10.1371/journal.pone.0209665 (PMC6353089; doi:10.1371/journal.pone.0209665)

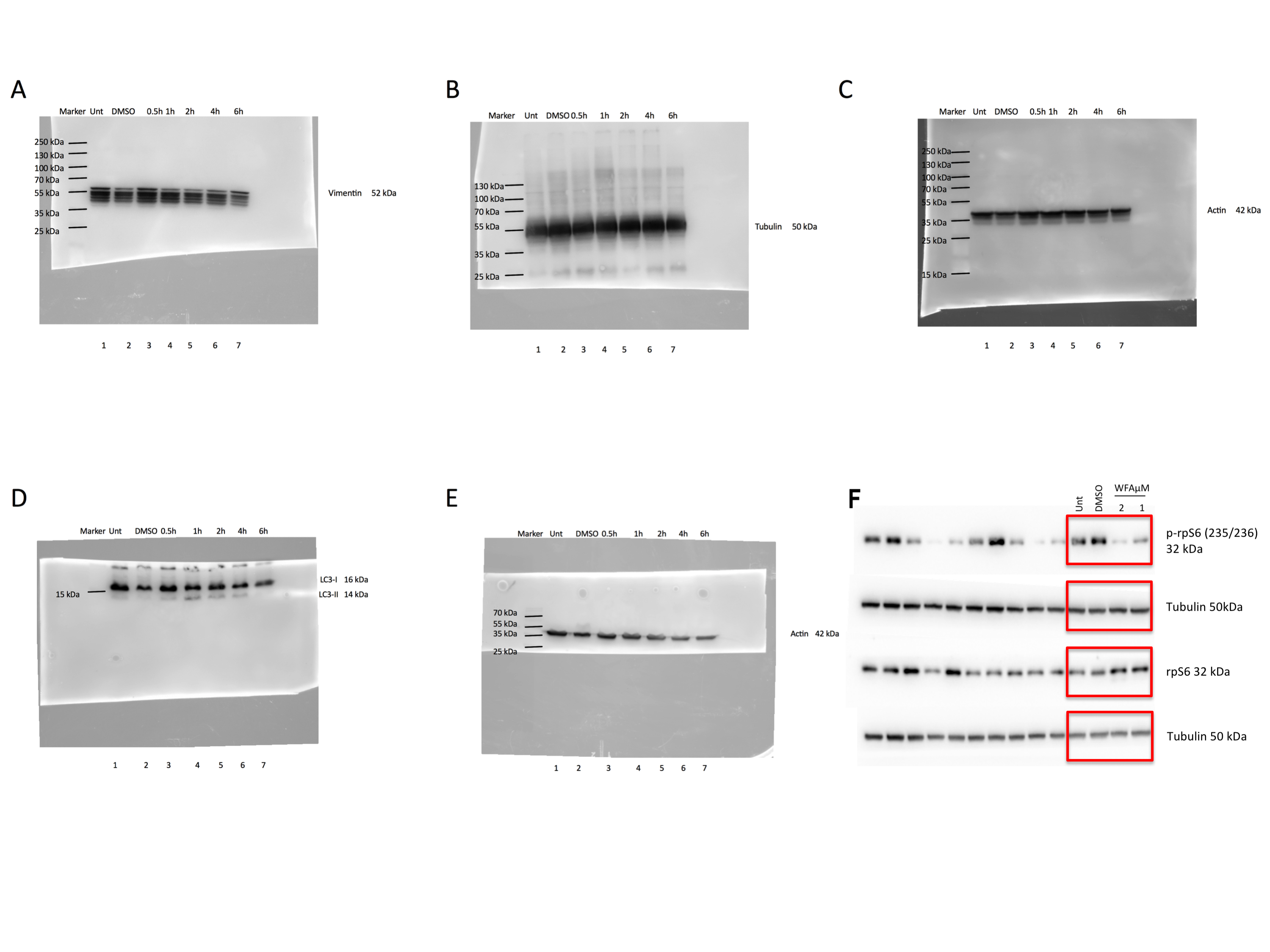

Supplement: S1 Fig — Uncropped immunoblots of vimentin (A), tubulin (B) and actin (C) corresponding to (Fig 1B); LC3-I/LC3-II (D) and actin (E) corresponding to (Fig 2A); and p-rpS6 (235/236), total rpS6 and tubulin (F) corresponding to (Fig 6A). Red boxes indicate where images were cropped. (TIFF) [file pone.0209665.s001.tiff]

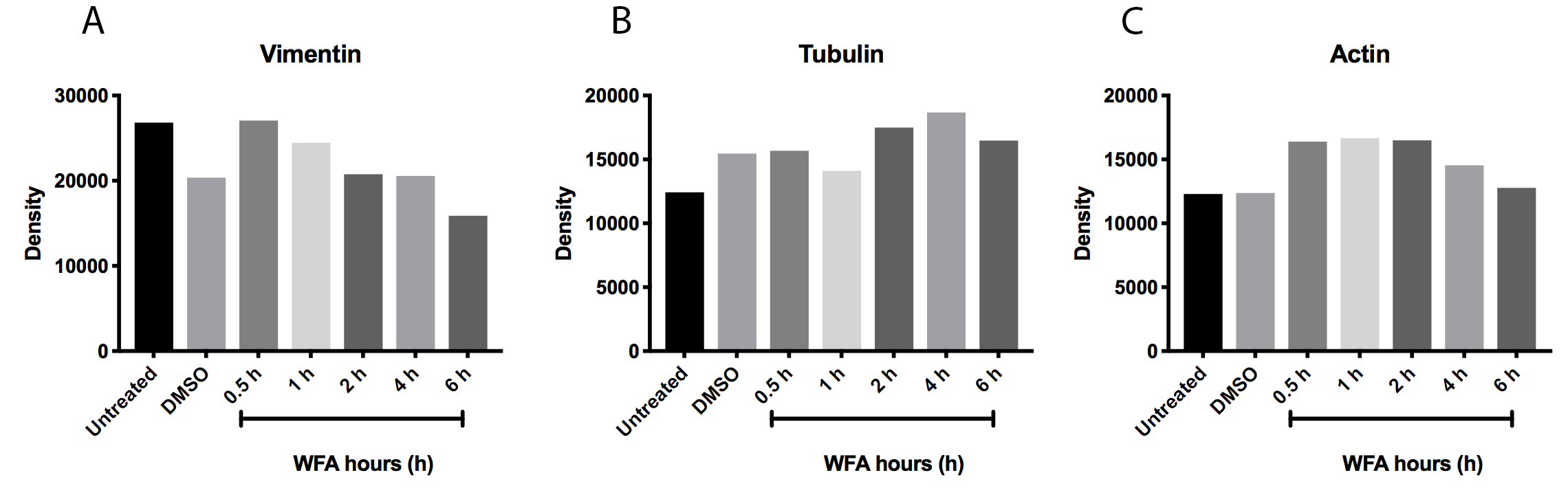

Supplement: S2 Fig — The relative intensity of vimentin (A), tubulin (B) and actin (C) was measured using Image J software. (TIF) [file pone.0209665.s002.tif]

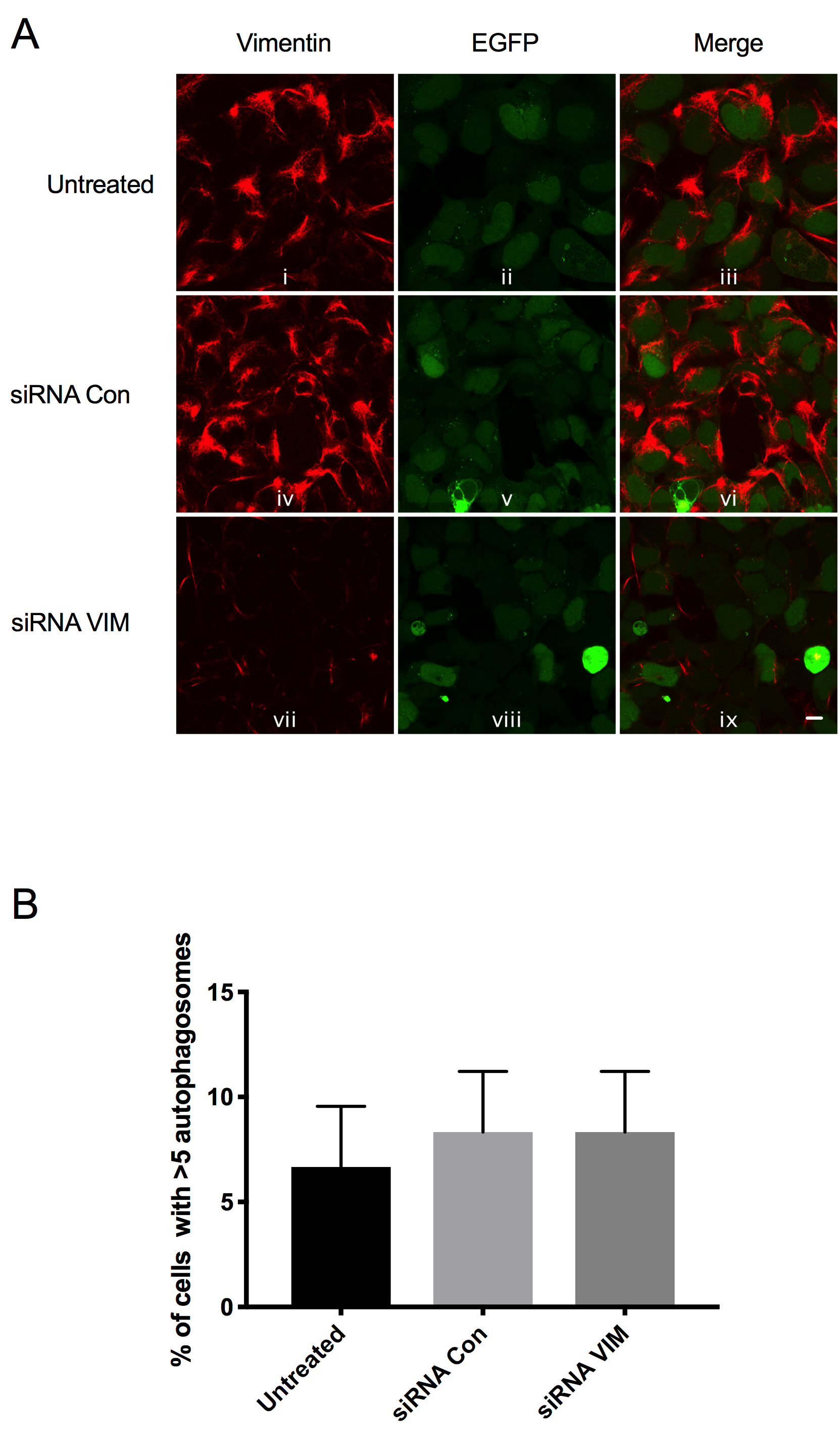

Supplement: S3 Fig — A) Immunofluorescence analysis of endogenous vimentin (red) and autophagosomes (green) in HEK293 GFP-LC3 cells treated for 48 h with 200 nM of human VIM or 200 nM human Non-targeting siRNA and compared to untreated cells. Scale bars are equal to 10 μm. Representative images are shown. B) Quantification of cells exhibiting >5 autophagosomes after 48 h of siRNA treatment and compared to untreated cells. (TIFF) [file pone.0209665.s003.tiff]

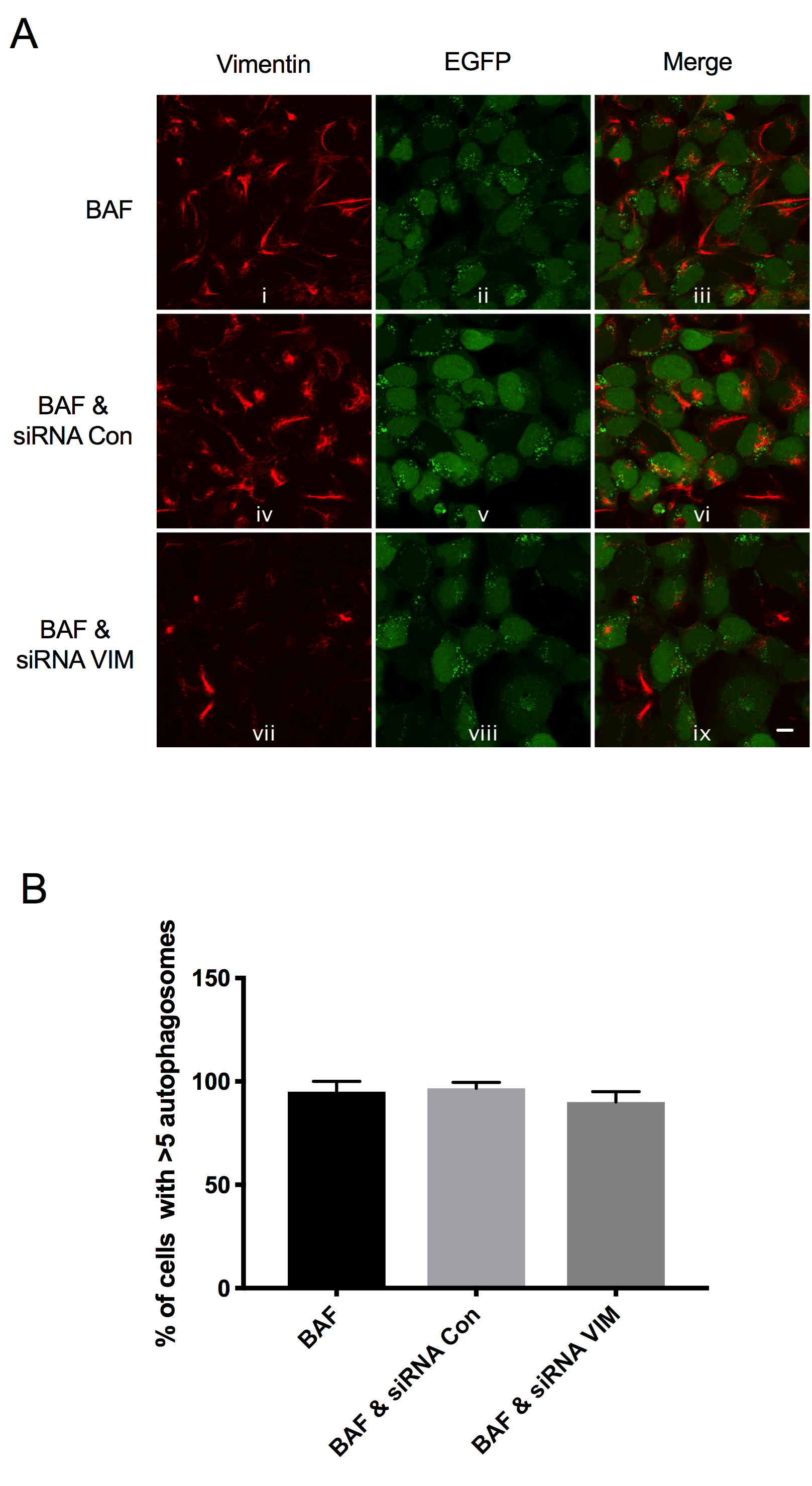

Supplement: S4 Fig — A) Immunofluorescence analysis of endogenous vimentin (red) and autophagosomes (green) in HEK293 GFP-LC3 cells treated for 48 h with 200 nM of human VIM or 200 nM human Non-targeting siRNA followed by BAF for 6 h and compared to BAF only treated cells. Scale bars are equal to 10 μm. Representative images are shown. B) Quantification of cells exhibiting >5 autophagosomes after 48 h of siRNA and BAF treatment. (TIFF) [file pone.0209665.s004.tiff]

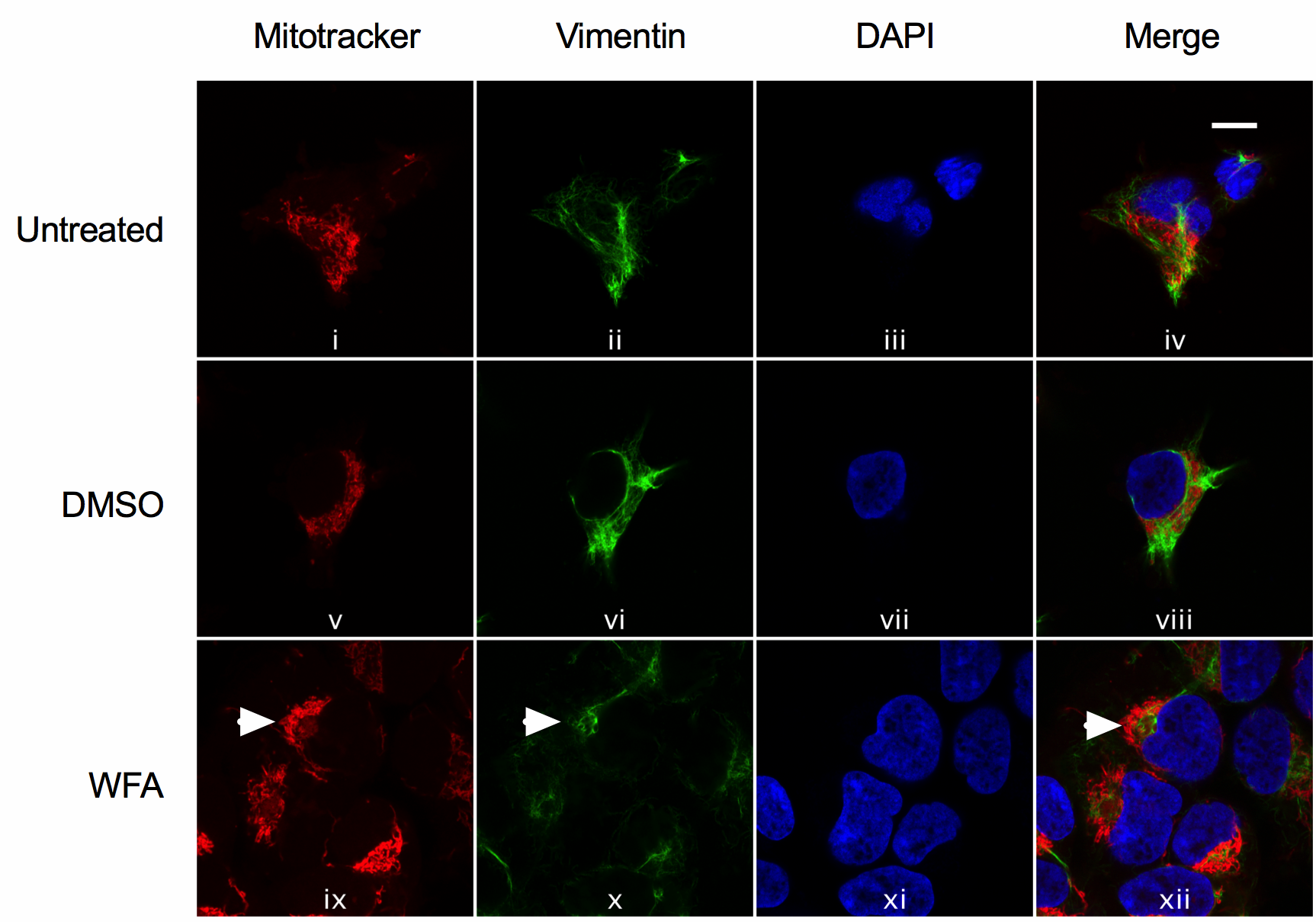

Supplement: S5 Fig — Immunofluorescence analysis of endogenous vimentin (green) and mitochondria (Mitotracker red) in HEK293 cells treated for 6 h with 1.5 μM of WFA, DMSO and compared to untreated cells. Cell nuclei were stained with DAPI (blue). Scale bars are equal to 10 μm. Representative images are shown. (TIFF) [file pone.0209665.s005.tiff]
